# Endophytic Colonization by the Entomopathogenic Fungus *Beauveria Bassiana* Affects Plant Volatile Emissions in the Presence or Absence of Chewing and Sap-Sucking Insects

**DOI:** 10.3389/fpls.2021.660460

**Published:** 2021-07-26

**Authors:** Natalia González-Mas, Fernando Gutiérrez-Sánchez, Araceli Sánchez-Ortiz, Luca Grandi, Ted C. J. Turlings, José Manuel Muñoz-Redondo, José Manuel Moreno-Rojas, Enrique Quesada-Moraga

**Affiliations:** ^1^Departamento de Agronomía, Escuela Técnica Superior de Ingeniería Agronómica y de Montes (ETSIAM), Universidad de Córdoba, ceiA3, Campus Rabanales, Córdoba, Spain; ^2^Department of Food Science and Health, Andalusian Institute of Agricultural and Fisheries Research and Training (IFAPA), Ctr Venta del Llano, Jaén, Spain; ^3^Fundamental and Applied Research in Chemical Ecology (FARCE Lab), Institute of Biology, University of Neuchâtel, Neuchâtel, Switzerland; ^4^Department of Food Science and Health, Andalusian Institute of Agricultural and Fisheries Research and Training (IFAPA), Alameda del Obispo, Córdoba, Spain

**Keywords:** endophyte, volatile organic compounds, plant-mediated interaction, *Spodoptera littoralis*, *Spodoptera frugiperda*, *Aphis gossypii*, cotton, melon

## Abstract

Entomopathogenic fungi are gaining acceptance in Integrated Pest Management (IPM) systems as effective and environmental safety biological control agents to protect a great variety of crops against pest insects. Many of these insect-pathogenic fungi can establish themselves as endophytes and thereby may induce the plant immune system. The activation of plant defenses by the fungal endophytic colonization can have a direct impact on herbivores and plant pathogens. An integral component of many plant defense responses is also the release of volatile organic compounds, which may serve as an indirect defense by attracting the natural enemies of herbivores. Here we investigated the effect of endophytic colonization by the entomopathogenic fungus *Beauveria bassiana* on the volatile emission by melon and cotton plants, either unharmed or after being damaged by sap-sucking aphids or leaf chewing caterpillars. We found that when the plants are colonized by *B. bassiana* they emit a different blend of volatile compounds compared to uncolonized control plants. Some of the emitted compounds have been reported previously to be released in response to herbivory and have been implicated in natural enemy attraction. Several of the compounds are also known to have antimicrobial properties. Therefore, endophytic colonization by *B. bassiana* might help to not only direct control insect pests but also increase the resistance of plants against agronomically important pests and phytopathogens.

## Introduction

Entomopathogenic fungi represent a large group of fungal species whose special feature is that they naturally infect and control insect populations (Lovett and St. Leger, 2017). They are gaining acceptance in Integrated Pest Management (IPM) systems, where they can serve as effective and environmental safety biological control agents of a great variety of crop pests (Zimmermann, 2007; Lacey and Shapiro-Ilan, 2008; Lacey et al., 2015; Quesada-Moraga, 2020). Besides their main habitats, soil and insect cadavers, they have recently been found to establish interesting plant associations (Meyling and Eilenberg, 2007; Vega et al., 2009; Quesada-Moraga, 2020). They can be found in the phylloplane, rhizosphere and as endophytes (Meyling and Eilenberg, 2006; Vega et al., 2008; Hu and Bidochka, 2019; Quesada-Moraga, 2020). These recent discoveries have been exploited in biological control, protecting systemically the plants from chewing and sap-sucking insects (Resquín-Romero et al., 2016; Garrido-Jurado et al., 2017; Jaber and Ownley, 2018; Vega, 2018). Moreover, these endophytic colonizations often have the additional benefit of protecting plants against several phytopathogens and promoting plant growth (Jaber and Ownley, 2018; Vega, 2018; Dara, 2019). This is explained by the fact that endophytes induce the plant immune system and activate plant defenses, as has been confirmed by different physiological plant changes observed in several studies (Gualandi et al., 2014; Shrivastava et al., 2015; Rondot and Reineke, 2019). An integral component of many plant defense responses is the production and release of volatile organic compounds (VOCs) in response to insect attack (Dicke and Baldwin, 2010; Heil, 2014; Turlings and Erb, 2018), but also pathogen infections (Ton et al., 2007; Howe and Jander, 2008; Sobhy et al., 2017; Hijri et al., 2018). In fact, certain endophytic fungi have been reported to emit VOCs that promote growth and influence the defense responses of their host plants (Strobel et al., 2011; Li et al., 2014; Shikano et al., 2017).

Several studies have demonstrated the plant-mediated effects of endophytic colonization by entomopathogenic fungi on insects and their natural enemies (Akutse et al., 2014; Gathage et al., 2016; Sword et al., 2017; Jaber and Araj, 2018; González-Mas et al., 2019a,b), but little is known about the identity and importance of volatiles that are directly emitted by the fungus or result from the endophytic colonization (González-Mas et al., 2019b; Moloinyane and Nchu, 2019; Fingu-Mabola et al., 2020). A recent study looked at the volatiles emitted by leaves from melon plants that had been inoculated with different strains of the entomopathogenic fungi *Beauveria bassiana* (Bals.) Vuill. or *Metarhizum brunneum* (Petch) (Ascomycota: Hypocreales) (González-Mas et al., 2019b). When these plants were subsequently infested by aphids, they showed qualitative and quantitative differences depending on the entomopathogenic fungus that had colonized the leaves, which in all cases were different from control plants (González-Mas et al., 2019b). Whether the spectrum of volatile compounds changes when plants are endophytically colonized by entomopathogenic fungi is as yet unknown. Likewise, it remains unknown if these differences in the volatile profile of endophytically colonized plants is also observed when entomopathogenic fungi colonized other plant families or if the kind of emitted volatile changes when they are infested by insects with chewing feeding behavior. If so, this could have important consequences for the plants' interactions with their biotic environment.

In the current study, we addressed the effect of the endophytic colonization by the entomopathogenic fungus *B. bassiana* on the volatile emission at different time points after colonization of melon and cotton plants, and how these emissions further changed after the plants were damaged by sap-sucking aphids and leaf chewing caterpillars. We used a non-targeted approach based on metabolomic techniques and multivariate statistical analyses to reveal the potential volatile markers emitted after the endophytic colonization.

## Materials and Methods

### Biological Material: Plants, Insect Populations, and Fungal Strain

Melon seeds (*Cucumis melo* L. var. Galia) were surface-sterilized in 2% sodium hypochlorite (Sigma-Aldrich, MO, USA) for 2 min, rinsed twice with sterile Mili-Q water and dried in a laminar-flow hood under sterile conditions. The soil substrate (Floragard, Germany) was also sterilized twice in an autoclave for 20 min at 121°C with a 24-h interval between each sterilization process. Surface-sterilized seeds were germinated in 400 ml pots containing a mixture of equal parts of vermiculite (No. 3, Asfaltex S.A., Barcelona, Spain) and the sterilized soil substrate. Germinated seeds were maintained in an environmental chamber under controlled conditions: 25 ± 2°C and a 16-h light: 8-h dark regime. A nutritional complex of 20:20:20 (N:P:K) Nutrichem 60 fertilizer (Miller Chemical & Fertilizer Corp., PE, USA) was added to the irrigation water at a rate of 1 g l^−1^ three times a week. Wild cotton seeds (*Gossypium hirsutum* L.) were collected in 2016 from a wild population in Puerto Escondido (Oaxaca, Mexico). Most research on direct defenses in cotton comes from studies with domesticated varieties, and this could be the cause of the different volatiles found in this study. It has been observed that cotton emissions are time dependent (Loughrin et al., 1994) starting with constitutive and then moving to also *de novo* inducible volatiles and DMNT and TMTT belong to the second category. These seeds were scratched with sandpaper to improve germination and pregerminated on cotton wool moistened with sterile water. After that, germinated seeds were transferred to 200 ml pots using sterilized soil substrate (COOP, Switzerland) and maintained in an environmental chamber set at 23°C and a 16:8 h light:dark regime. Plants were fertirrigated three times a week.

Aphid colonies were obtained from a virus-free laboratory population of *Aphis gossypii* Glover (Homoptera: Aphididae) provided by the Institute of Agricultural Sciences (ICA) CSIC (Madrid, Spain) for use in the assays. The aphids were maintained in rearing cages on melon plants (*Cucumis melo* L. var. Galia) for several generations in an environmental growth chamber under controlled conditions: 25 ± 2°C, 16:8 h light:dark regime, and 70% RH. For each experiment, newly emerged apterous adult females (24–72 h after last molt) were collected from the rearing cages using a camel-hair brush and used immediately in experiments. The caterpillars *Spodoptera littoralis* (Boisduval) and *Spodoptera frugiperda* (Smith) (Lepidoptera: Noctuidae) were reared as described by Maag et al. (2014). Briefly, *S. littoralis* larvae were reared from eggs provided by Syngenta (Stein, Switzerland). The eggs were kept in an incubator (25°C and LD 16: 8 h) and, after emergence, larvae were placed on a wheat germ-based artificial diet at room temperature. Larvae of *S. frugiperda* were obtained from a colony established at the University of Neuchâtel. They were reared on a chickpea flour-based artificial diet in plastic boxes under artificial light conditions and at ambient temperature. For the experiments, second-instar larvae were collected and placed on the cotton plants to induce the production of volatiles. Although differences exist in the quantities of plant volatiles that they may induce, both species induce a volatile blend of similar composition. Also, application of regurgitant of both species to wounded plants, as a means of artificial induction of defenses, results in the emission of similar blends of volatiles (De Lange et al., 2020).

*Beauveria bassiana* strain EABb 01/33-Su was used in all the bioassays. The endophytic ability of this strain when inoculated onto melon and cotton plants has been demonstrated previously, as has its ability to cause mortality in chewing and sap-sucking insects when endophytic (Resquín-Romero et al., 2016; Garrido-Jurado et al., 2017; González-Mas et al., 2019b). EABb 01/33-Su was originally isolated from soil from El Bosque (Cádiz) and deposited at the University of Córdoba Entomopathogenic Fungi Collection, Córdoba, Spain and at the Spanish Collection of Culture Types (CECT), University of Valencia, with accession n°. CECT 21149. Nucleotide sequences for the ITS and mtDNA intergenic regions of EABb 01/33-Su can be found in the Gen-Bank database (EF115310 and FJ972969 for the ITS region; FJ973025 for intergenic region nad3-atp9; and FJ972914 for the intergenic region atp6-rns). For all bioassays, the EABb 01/33-Su strain was grown on potato dextrose agar (PDA) in Petri dishes for 15 days at 25°C in darkness. A cellophane film was placed on the agar prior to inoculation to prevent nutrients transferring to the conidial suspension at the time of harvest. Conidial suspensions were prepared by scraping conidia from the dishes into an aqueous sterile solution of 0.01% Tween 80. The resulting conidial suspension was filtered through several layers of sterile cheesecloth to remove mycelia, and sonicated for 5 min to homogenize the inoculum. Conidial concentrations were determined using a haemocytometer and appropriate dilutions made in 0.01% Tween 80 to achieve a concentration of 10^8^ conidia ml^−1^ for experiments. Prior to experimentation conidial viability was determined on liquid Czapek-Dox broth plus 1% (w/v) yeast extract medium and only suspensions with >97.0% germination after 24 h were used.

### Inoculation of Plants With Entomopathogenic Fungi and Verification of Endophytic Colonization

For each experiment, replicate groups of five-leaf-stage melon and cotton plants were treated. Two leaves per plant were sprayed with 2 ml of the fungal suspension with an aerograph 27085 (piston compressor of 23 l min^−1^, 15–50 PSI and a 0.3 mm nozzle diameter, China). During application the remaining leaves were protected from the spray with a transparent plastic sheet and remained uninoculated. After inoculation, all plants were covered by another plastic sheet to promote fungal growth for 24 h. Control plants were treated in the same way but only sprayed with sterile water with 0.01% Tween 80. Before the plants were transferred to the vessels to analyze their emitted volatile profile, the sprayed leaves and the soil were covered with aluminum foil to avoid the emission of volatiles from the fungal inoculum from the surface of the leaf and from the soil. This cover also avoided the insect access to the sprayed leaves.

To confirm endophytic colonization, samples of leaves from fungal sprayed and control plants were collected when each experiment had finished to avoid damaging the plant and triggering plant defenses, which may have confounded the results. Inoculated and uninoculated leaves were sampled from each replicate plant, surface-sterilized with 1% NaOCl for 2 min, rinsed twice in sterile distilled water, and dried on sterile filter paper. Sections of ~2 cm^2^ were cut with a sterile scalpel from each leaf and plated out independently in Petri dishes containing selective culture medium to determine the percentage colonized endophytically; the medium contained: 20 g of Agar Sabouraud Glucose Chloramphenicol (Cultimed Panreac, Spain), 500 mg l^−1^ streptomycin sulfate (Sigma-Aldrich Chemie, China), 500 mg l^−1^ ampicillin (Intron biotechnology, China) and 500 mg l^−1^ dodine 65 WP (Barcelona, Spain). We also plated out the last rinse water from each leaf separately to confirm the effectiveness of the surface-sterilization procedure. All plates were incubated at 25°C in darkness until fungal growth was observed.

### Analysis of Volatile Compounds From Caterpillar-Infested, Endophytically-Colonized Plants

Wild cotton plants were grown and treated as described above. Volatiles emitted by control and treated plants were trapped at three different time points: (T0) Immediately after the treatment with the fungal suspension or the control solution, when the fungal colonization had not yet started; (T1) 48 h after the treatment, when the endophytic colonization was established in the plants treated with the fungal suspension; (T2) 72 h after the treatment and 24 h after the plants were damaged by caterpillars. The leaf damage treatment to induce plants to emit plant volatiles consisted in exposing each cotton plant to 10 s-instar larvae of *S. frugiperda* or *S. littoralis* were placed on each plant. Plants were infested 48 h after fungal treatment, once the collection of volatiles at T1 time was performed and the evening before the day collecting at T2 time.

These insects have been considered representative for assessing plant-induced responses to herbivory by chewing insects as they have been shown to induce chemical defenses in both wild and cultivated cotton (Chappuis and Egger, 2016).

Plants were placed in hermetic vessels and a Hayesep-Q adsorbent filter trap (25 mg, 80–100 mesh; Sigma, Switzerland) was attached to the horizontal port at the top of each odor source vessel. Volatile collection was performed during 1 h as adapted from Turlings et al. (1998). Purified air entered the bottles at a rate of 1 L min^−1^ inflow, and air carrying the volatiles was pulled through each trap at a rate of 0.9 L min^−1^ outflow. Subsequently, filters were eluted with 100 μL dichloromethane (Super solvent; Merck, Dietikon, Switzerland), the elute was then spiked with 10 μL internal standards solution (n-octane and nonyl-acetate, [20 ng/μL] each). Samples were analyzed on a gas chromatograph (GC Agilent 6890N) coupled to a mass spectrometer detector (MSD Agilent 5973). A 2 μL aliquot of each sample was injected in pulsed splitless mode onto an Agilent HP-5MS column (30 m length × 250 μm diameter and 0.25 μm film thickness). After injection, temperature was maintained at 40°C for 3 min, increased to 100°C at a rate of 8°C per min and subsequently to 200°C at a rate of 5°C per min followed by a post run of 3 min at 250°C. Helium was used as carrier gas and kept at constant flow of 1.1 ml min^−1^. Volatile compounds were identified at different levels of confidence on the basis of the proposed minimum criteria defined by the metabolomics standard initiative (Goodacre et al., 2007). Metabolites were definitive annotated (level 1) by comparing the MS spectra and linear retention index (LRI) against available standards purchased from Sigma-Aldrich-Merck [heptane, (*E*)-2-hexenal, (Z)-tridec-3-ene, acetic acid; hex-4-en-1-ol, ethyl heptanoate, nonanal, decanal, 2-ethylhexan-1-ol, benzaldehyde, 3,7-dimethylocta-1,6-dien-3-ol (linalool)]. Putative (or tentative) identifications (levels 2 and 3) were considered by comparing the MS spectra and LRI against existing databases. Metabolites for which MS spectra and LRI were not available in bibliography were labeled as “unknown” (level 4). Volatile compounds increases were expressed as fold-change.

There were six replicate plants for each treatment and each plant was measured at the abovementioned time points. The experiment was repeated three times using six new replicate plants, caterpillars and fungal inoculum each time, for a total of 18 plants two of these repetitions were performed using *S. frugiperda* larvae while the last one was performed with *S. littoralis*.

### Analysis of Volatile Compounds From Aphid-Infested, Endophytically-Colonized Plants

Melon plants were grown and treated as described above. In this assay, volatiles emitted by control and treated plants were also trapped at three different time points: (T0) Immediately after the treatment with the fungal suspension or the control solution, when the fungal colonization had not yet started; (T1) 48 h after the treatment, when the endophytic colonization had established in the fungal treated plants; (T2) 7 days after the treatment and 6 days after the plants were damaged by aphids, a last sampling was realized 21 days after the treatment and 20 days after the plants were damaged by the aphids (T3). Each melon plant was infested with ten adult *A. gossypii* aphids. Plants were infested 48 h after fungal treatment, once the collection of volatiles at T1 time was performed. These insects have been considered representative for assessing plant-induced responses to herbivory by sap-sucking insects due to they have been shown to induce chemical defenses (Hegde et al., 2012).

Plants were transferred in a vessel covered by a plastic bag and placed in a bath heated to 30°C and allowed to equilibrate for 10 min. Volatile compounds were collected during 50 min using a solid-phase microextraction (SPME) fiber (50/30 μm DVB/CAR/PDMS Stableflex 23Ga, Autosampler, SUPELCO, Bellefonte, PA, USA) that was introduced into the top of the plastic bag. Once the volatiles were collected, they were analyzed using a High-Resolution Gas Chromatograph with Triple Quadrupole systems Mass Spectrometry (HR-GC-TQ MS) (Bruker model Scion 456-GC-TQ MS system; Bruker, Massachusetts, USA). Method parameters were set based on previous works (Sánchez-Ortiz et al., 2013; González-Mas et al., 2019b). Desorption of volatile compounds was done directly into the GC injector at 250°C for 5 min. A Supelcowax 10 capillary column (30 m × 0.25 mm, 0.25 μm, Sigma-Aldrich Co. LLC) was used with the following parameters: Helium as the carrier gas; injector was set at 250°C; column held for 5 min at 40°C and then the temperature programed to increase at a rate of 4°C min^−1^ until it reached 200°C.

The MS method was as follows: EI mode (70 eV), ion source and transfer line temperatures were all fixed at 250°C. Mass spectra were obtained in full scan mode in the 30–250 mass-to-charge ratio range at a scanning speed of 7 scan/s. Chromatograms and spectra were recorded and processed using the Bruker MS Workstation version 8.2 (Bruker, Massachusetts, USA). Volatile compounds were identified at different levels of confidence on the basis of the proposed minimum criteria defined by the metabolomics standard initiative (Goodacre et al., 2007). Metabolites were identified as describe above for cotton plants.

There were five replicate plants for each treatment and each plant was measured at the abovementioned time points. The experiment was repeated twice using five new plants, aphids and fungal inoculum each time, for a total of 10 plants.

### GC-MS Pre-processing

Raw chromatograms were converted into international ANDI file format (.cdf) by means of the open source software OpenChrom. Deconvolution of the data was carried out using PARAllel FACtor analysis2 (PARAFAC2), since a minimal number of hyperparameter have to be set and it allows to handle complex chromatograms displaying coelutions, low signal-to-noise (S/N) ratios and shifts in retention times (Amigo et al., 2008). Chromatograms were divided in subintervals and independent models were performed to reduce the complexity. A total of 8 components were considered for each model, and the optimum number was optimized on the basis of the core consistency, comparison of the resolved mass spectral against raw profiles, retention times, distribution of residuals and model fit (%). Afterwards, all the deconvoluted peaks were manually inspected. The PARAFAC2 mass resolved mass spectrum was compared against the NIST (version 2.0, NIST, USA) for putative identification as described in the previous section. The freely available software PARADISe v.3.88 was used for this deconvolution procedure.

### Statistical Analysis

Data on volatile compounds were analyzed using Kolmogorov–Smirnov and Levene's tests to evaluate parametricity of the data (normality and homogeneity of variance), and variables failing one of these linear model assumptions were Box-Cox transformed. Then, analysis of variance (ANOVA) was applied at a significance level of 0.05. The Tukey's HSD test was used for pairwise comparisons. Mixed models were analyzed with proc GLIMMIX-SAS 9.3; Pearson Chi-square, Kaplan Meier and linear models were analyzed with SPSS 24.0. Due to the nature of this study, we may expect a high intrinsic variability between plant replicates. Therefore, we first evaluated the variability in the repetitions to decide whether to pool them or evaluate it by separate to avoid misleading results. On this basis, the data from melon plants were pooled, since statistically significant repetition-related differences were not observed. However, the data from cotton plants were more heterogeneous across the three repetitions and we decided to separately assess each experiment.

Multivariate data analysis was performed to unravel the structure of the GC-MS data. Principal Component analysis (PCA) was built to look for trends and variability patterns. Subsequently, the most relevant metabolites (potential markers) were selected by means of an iterative procedure based on Variable Importance in Projection (VIP) scores from Partial Least Squares Discriminant Analysis (PLS-DA) and described in Muñoz-Redondo et al. (2019). Afterwards, the most discriminative metabolites were used to fit PLS-DA models double cross-validated using k-fold resampling method in the outer loop and leave-one-out in the inner loop (Szymańska et al., 2012). The PLS-DA models were validated by means of a permutation test based on balanced error rate (BER), number of misclassified (NMC) and area under the operating receiver curve (AUROC). We used *N* = 1,000 since it was large enough to sample the tails of the distribution and to attain a *p*-value up to 0.001.

When there was no statistically significant difference in the results from the repetitions on which each experiment was done the data were pooled, analyzed together, and presented as a single table. In addition, when no statistically significant difference in the results from the different sampling timepoints at the volatiles were measured, the data were pooled as well.

## Results

### Endophytic Colonization of Melon and Cotton Plants

Microbiological techniques confirmed endophytic fungal colonization by the entomopathogenic fungus *B. bassiana* in 100% of the leaves that had received fungal sprays directly, but in only 20–40% of the unsprayed leaves (33.33 ± 17.64% for cotton plants and 56.67 ± 0.00% for melon plants) from plants that also had sprayed leaves. Individual plants were considered as positive for endophytic colonization based on detection of fungus within any of the leaves sampled on that plant. On the contrary, *B. bassiana* colonization was not observed in any of the samples from the control plants.

### Effect of Endophytic Colonization on Volatile Compounds Emitted From Caterpillar-Infested Cotton Plants

**Figure 1:**
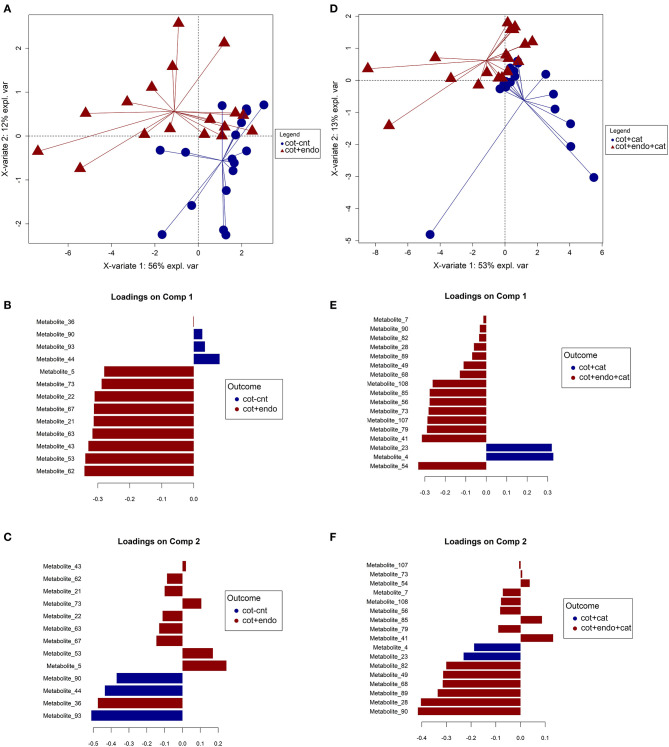
Graphical outputs of the two Partial Least Squares Discriminant Analyses (PLS-DA) performed on volatile compounds emitted from endophytically colonized and control cotton plants before **(A–C)** and after **(D–F)** caterpillar infestation. **(A)** Scores plot for the component 1 and 2 (X-variate 1 and X variate 2); **(B)** loading contribution barplot on component 1; **(C)** loading contribution barplot on component 2. **(D)** Scores plot for the component 1 and 2 (X-variate 1 and X variate 2); **(E)** loading contribution barplot on component 1; **(F)** loading contribution barplot on component 2. Color indicates the class for which the compound has a maximal mean value. Bar length represents the multivariate regression coefficient with either a positive or negative sign for that particular feature on each component, i.e., the importance of each variable in the model. Cot-cnt: control at T0+T1, cot+cat: control and caterpillar-infested at T2, cot+endo: treated at T0+T1, cot+endo+cat: treated and caterpillar-infested at T2.

A total of 101 volatile compounds were tentatively identified (Supplementary Table 1) including plant-derived compounds, herbivore-induced volatiles and fungal-derived compounds. To assess the impact of the endophytic colonization on volatile compounds emitted from caterpillar plants before and after infestation, two PLS-DA models were independently fitted to differentiate (i) control plants (cot-cnt) from treated plants (cot+endo) and (ii) infested control plants (cot+cat) from endophytic colonized infested plants (cot+endo+cat). An iterative procedure based on partial least squares discriminant analysis (PLS-DA) was used to select for the most relevant metabolites, which were further used to construct a final PLS-DA model shown in Figure 1. Satisfactory classification performances were achieved for both models optimized for four components with a BER of 0.18 ± 0.05 (i) and 0.16 ± 0.04 (ii), as it is shown in Supplementary Table 2. The main variation sources in the samples were explained by the first two principal components. In model (i) the first two principal components explained around the 68% of the total variance of samples. In component 1, the volatile metabolites 62 (unknown), 53 (unknown), 43 (unknown), 63 (2,7,10-trimethyldodecane), 21 (4-methyloctane), 67 (2,6-dimethyldecane), 22 [(E)-hex-2-en-1-ol], 73 (isothiocyanatocyclohexane), and 5 (3-chloro-3-methylpentane) were selected as potential markers for the endophytic colonization before the infestation due an overall increase in their concentrations (Figure 1). The different starting levels and the high variability of the volatiles emitted by caterpillar plants in the three experimental assays did not allow to clearly establish this behavior using the ANOVA statistic (Supplementary Table 1). Therefore, these results support the use of multivariate beside the univariate statistical techniques to reveal trends in complex datasets. Other relevant volatiles selected on component 2 were the metabolites 93 [2,2,6-trimethyl-9,10-dioxatricyclo(6.2.2.01,6)dodecan-3-ol], 44 (5-ethyl-2,2,3-trimethylheptane), and 90 [(3-hydroxy-2,4,4-trimethylpentyl)2-methylpropanoate], displaying higher levels in treated plants, and metabolite 36 [2,2-dimethyl-3-methylidenebicyclo(2.2.1)heptane], which was found to be increased in control plants. The lower explained variance on component 2 (12% compared to the 56% of component 1), suggests slighter changes on these compounds due to the effect of the endophytic colonization before the infestation. Meanwhile, the impact of the endophytic colonization after infestation of caterpillar plants was addressed in model (ii). The first two principal components explained around the 66% of the total variance found in samples, which made it possible to separate cot+cat from cot+endo+cat plants. An overall increase of the selected markers, which were quite different to the former approach, was again observed in treated plants. Only the volatile metabolite 90 was selected in both comparisons, but displaying an opposite trend, as it can be seen in component 2 of both models (Figure 1). This compound diminished its concentration in cot+endo plants before infestation but increased in the same samples after the infestation. The most important volatiles selected on component 1 were the metabolites 54 [(5R)-1-methyl-5-prop-1-en-2-ylcyclohexene), 41 (benzaldehyde), 79 (3-[(E)-dodec-2-enyl]oxolane-2,5-dione), 107 (diethyl benzene-1,2-dicarboxylate), 73 (isothiocyanatocyclohexane), 56 (unknown)], 85 (cyclohex-3-en-1-ylbenzene) and 108 [(2Z,13E)-octadeca-2,13-dien-1-ol] with overall increased in TT-P plants and metabolites 4 (3-chloro-3-methylpentane), 23 (1,1,2-trimethyl-3-(2-methylpropyl)cyclopropane] and with overall lower levels in these samples. In the same line than model (i), the wide variance explained by component 1 highlights their main contribution to discriminate both labeled groups. Meanwhile, markers selected on component 2 corresponded to metabolites 90 [(3-hydroxy-2,4,4-trimethylpentyl) 2-methylpropanoate], 28 (heptanal), 89 (2-Methylpropyl 3-hydroxy-2,2,4-trimethylpentanoate), 68 (2-ethenyl-1,1-dimethyl-3-methylidenecyclohexane), 49 (octanal), and 82 (nonyl acetate), which were found in higher overall concentrations in cot+endo+cat samples.

In addition, they have been identified several compounds usually found in cotton plants. For example, (Z)-hex-3-en-1-ol (metabolite 18), 2,6,6-Trimethylbicyclo[3.1.1]hept-2-ene (metabolite 33) known as “α-pinene,” 1H-indole (metabolite 78), (1R,4E,9S)-4,11,11-Trimethyl-8-methylidenebicyclo[7.2.0]undec-4-ene (metabolite 95), and (1E,4E,8E)-2,6,6,9-Tetramethylcycloundeca-1,4,8-triene (metabolite 97), known as “caryophyllene” and “α-caryophyllene or humulene” have been found by Sobhy et al. (2014) in *S. littoralis* damaged plants. 1H-indole (metabolite 78) has been showed to increase in infested plants, and endophytically colonized caterpillar infested plants emitted in highest quantities. Caryophyllene (metabolite 95) was found in higher contents in treated cotton plants than in the control, dramatically reducing the emission in endophytically colonized caterpillar infested plants. Finally, α-caryophyllene (metabolite 97) and decanal were found in general in less quantity in caterpillar-infested plants than in non-infested plants taking into account all the treatments. However, if non-infested are compared, endophytically colonized plants emitted in higher quantities than in the control ones.

It is noteworthy that the samples showed considerable differences among the three experimental assays. The endophytic colonization in the first and third assays impacted much stronger on several compounds, especially for metabolites 16 [(E)-hex-2-enal], 17 [(NZ)-N-(2-methylbutylidene)hydroxylamine], 18 [(Z)-hex-3-en-1-ol], 57 (3,7,7-trimethylbicyclo[4.1.0]hept-3-ene), 68 (2-ethenyl-1,1-dimethyl-3-methylidenecyclohexane), 78 (1H-indole), 87 (methyl anthranilate), 105 (isocaryophillene) and 107 (diethyl benzene-1,2-dicarboxylate) with increases ranging from 5 to more than 5000-fold the initial levels of the control plants (Supplementary Table 1). The contrary or soft variations of these compounds observed in the second experimental assay could have been prevented these metabolites to be selected as markers for the endophytic colonization during the variable reduction procedure. These differences could be due to in the assays they were used three different batches of plants grown in different time periods. In addition, despite no differences were found in other studies when the volatiles released by the two species of caterpillars were compared, it could have had an effect in this work due to the presence of the entomopathogenic fungal endophyte.

### Effect of Endophytic Colonization on Volatile Compounds Emitted From Aphid-Infested Melon Plants

**Figure 2:**
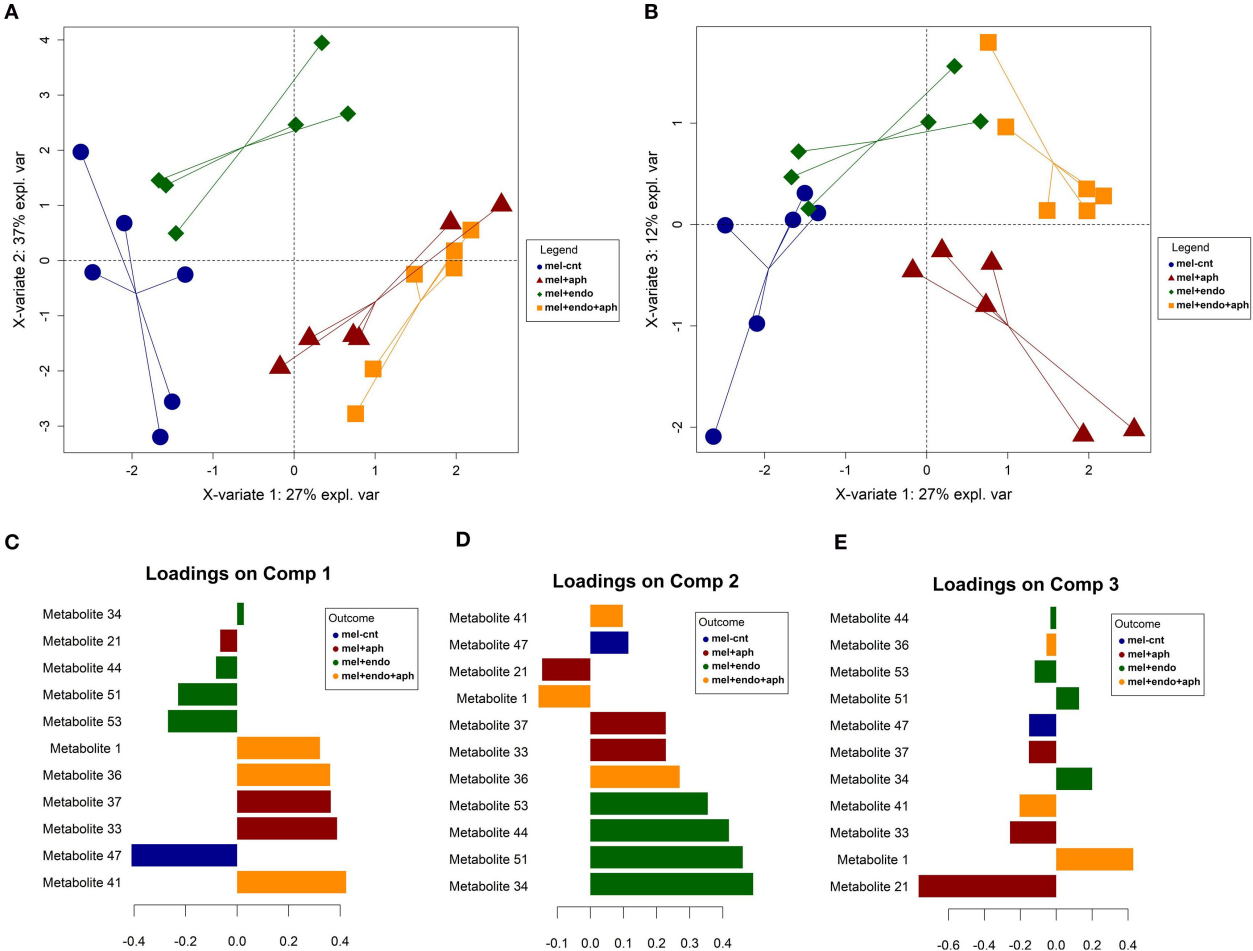
Graphical outputs of the Partial Least Squares Discriminant Analysis (PLS-DA) performed on volatile compounds emitted from endophytically colonized and control melon plants before and after aphid infestation **(A)** Scores plot for the component 1 and 2 (X-variate 1 and X variate 2); **(B)** Scores plot for the component 1 and 3 (X-variate 1 and X variate 3); **(C)** loading contribution barplot on component 1; **(D)** loading contribution barplot on component 2; **(E)** loading contribution barplot on component 3. Color indicates the class for which the compound has a maximal mean value. Bar length represents the multivariate regression coefficient with either a positive or negative sign for that feature on each component, i.e., the importance of each variable in the model. Mel-cnt: control at T0+T1, mel+aph: control and aphid-infested at T2+T3, mel+endo:treated at T0+T1, mel+endo+aph: treated and aphid-infested at T2+T3.

Just as for the cotton plants, to assess the impact of fungal colonization on the volatile emission of melon plants aphid-infested and non-infested, a wide range of volatile compounds in terms of its chemical nature were tentatively identified. In a similar fashion as the caterpillar-infested plants, the final models were fitted with the most relevant metabolites (Figure 2). Four clusters of samples were considered, corresponding to control (mel-cnt), control-infested (mel+aph), treated (mel+endo) and treated-infested (mel+endo+aph) and a model with a satisfactory balanced error rate (BER) of 0.14 ± 0.07 was obtained (Supplementary Table 4). Disclosing by groups, it was observed a slightly higher prediction accuracy for mel+endo+aph samples with a low mean error class of 0.12, due to wrong assignments to mel+aph samples during the cross-validation. While the worst prediction abilities were attained for the samples mel+aph (0.24 of mean class error) mainly driven by mislabeling as mel+endo+aph plants (Supplementary Table 4). The model was optimized for four components, which accounted for a high 87% of the total variability found in the plant samples. This high percentage of variability explained in the model supports the robustness of this study, taken into account the inherent variability in the volatile emission of different plants and the potential experimental errors. The subspace spanned by the first three principal components explained the most interesting information and revealed well-defined clusters of the target classes (Figure 2). A clear trend toward more positive values of the X-variate 1 (component 1) due to the infestation of the plants was observed, i.e., from mel-cnt to mel+aph and from mel+endo to mel+endo+aph. This trend was partially explained by an increase on levels of the metabolites 41 (2-butyloctan-1-ol), 33 (ethyl heptanoate), 37 (3-methyltridecane), 36 (hexadecan-4-yl 2,2,2-trifluoroacetate) and 1 (heptane) in mel+aph and mel+endo+aph. Variations on these compounds are expressed as foldchange in relation to the mel-cnt samples in the ANOVA of Supplementary Table 3. A strong increase for up to 15-fold was recorded for levels of the metabolite 41 after infestation of control plants. A similar response was observed in plants treated with the entomopathogenic fungus *B. bassiana* but even before to infestation. The metabolites 33 (ethyl heptanoate) and 36 (hexadecan-4-yl 2,2,2-trifluoroacetate) displayed the same behavior with a softer response, reaching no more than 2-fold the initial levels of the control plants. Aphid-infested plants gave rise to significant increases in Metabolite 37 (3-methyltridecane) which an average of three times over control plants. The behavior of metabolite 1 (heptane) was quite interesting, since its only significant variation along the experiment was observed in mel+endo-t3, growing up to 12-fold in this point (Supplementary Table 3; of note the high standard deviation due to a soft response in one plant assay). In addition, the trend noted on X-variate 1 was also driven by a strong decrease, ranging from 90 to 94%, on metabolite 47 (2-ethylhexan-1-ol) contents, which was particularly acute for the plants treated with *B. bassiana* (Supplementary Table 3). Component 2 revealed a different behavior between control and treated plants related to the infestation. While the controls did not show a clear variation on the direction of component 2, the plants treated with *B. bassiana* moved toward more negative values after infestation. This behavior was mainly explained by a decrease on certain metabolites. Specially, for metabolites 44 (unknown), 51 (oxolan-2-one) and 53 (phenyl carbamate), these decreases were of similar magnitude, between 30 and 50% in mel+endo+aph-t3 plants. The response of metabolite 51 in control and treated plants was similar, although the changes were softer in the first case. The rest of addressed metabolites displayed a reduction in its emission before aphids contact (mel-cnt and mel+endo) and a change of positive trends after aphid infested (mel+aph and mel+endo+aph) (Supplementary Table 3). Finally, component 3 explained new hidden information in previous components, which could be just related to the treatment effect of *B. bassiana* endophytic colonization, since both mel-cnt and mel+aph on one side and mel+endo and mel+endo+aph on the other side were between, but not within, separated. The metabolite 21 (2-ethyldecan-1-ol) was the main responsible explaining this behavior, since it was found to be decreased in plants treated with *B. bassiana* for around the 50% in mel+endo+aph-t3 compared to mel+aph-t3.

## Discussion

It is well-documented that microbes can significantly affect and contribute to the release of specific volatiles by plants (Pineda et al., 2010; Kanchiswamy et al., 2015), but to the best of our knowledge, ours is the first study to measure the volatile emissions from plants that have been colonized by an entomopathogenic fungus. We measure the emissions at different time points after fungal inoculation and determined how they changed after the plants were damaged by sap-sucking and chewing herbivores.

It has been proposed that the effectiveness of microbial control agents can be enhanced by manipulating or enhancing the release of VOCs, as this may boost the plants' repellence to insect herbivores and/or the attraction of other important biological control agents, such as entomophagous insects (Sword et al., 2017; Brilli et al., 2019; González-Mas et al., 2019a; Moloinyane and Nchu, 2019). In fact, several studies on endophytic entomopathogenic fungi have demonstrated that they can enhance insect plant resistance, but also a surprising increase in the plants' attractiveness to generalist predators (Vega, 2018; González-Mas et al., 2019a). A possibility is that the blend of volatiles emitted by endophytcally colonized plants could be involved in this attractiveness of plants for insects, though further research is needed to confirm this. A previous study on volatiles from processed leaves from melon plants found several changes in the blend of volatile emissions after a plant is colonized by entomopathogenic fungi and subsequently infested by aphids, in comparison to uncolonized aphid-infested plants (González-Mas et al., 2019b). The present study was motivated by the question whether what was observed using processed melon leaves is also true for intact plants. We studied this here for cotton, as well as melon plants. Results seem to indicate a regulatory effect specific on the pathway of biosynthesis of volatile compounds emitted by plants.

In general, in the assay with wild cotton plants considerably larger amounts of volatile compounds were detected as compared to melon plants, but it should be noted that different extraction methods were used. The wild cotton plants constitutively emitted various monoterpenes and sesquiterpenes such as myrcene, β-caryophellene and α-humulene, while the volatile blend of melon plants contained short chain aldehydes, alcohols, acids, acetates and their derivatives, as previously reported for this cucurbitaceous crop (Hou et al., 1997). Besides that, it was confirmed in the present research that colonization by the entomopathogenic fungus *B. bassiana* resulted in the emission of a different blend of volatile compounds as compared to control plants. In previous works it has been demonstrated that endophytic microorganisms and related plant strengtheners can strongly affect the herbivore-induced volatile blends and has been shown to render the plants considerably more attractive to natural enemies (Rostás and Turlings, 2008; D'Alessandro et al., 2014; Sobhy et al., 2018). One possible explanation for the changes in the volatile blends is that defense responses are triggered in fungally challenged plants, which involve volatile releases (Rivas-Franco et al., 2020). This has been observed for plants that were treated with methyl jasmonate or cis-jasmone, increasing the production of defense compounds and simultaneously changing the emission of VOCs (Brunissen et al., 2010; Sobhy et al., 2017).

Regarding cotton, several of the compounds that were found to be released in larger amounts after colonization by *B. bassiana* EABb 01/33-Su strain are known to attract natural enemies, which could explain the enhanced attractiveness of colonized plants to them observed in previous studies (González-Mas et al., 2019a), whereas it will need further research to determine if the volatiles released by endophytically colonized plants are in fact involved in attractiveness of plants for insects. Indeed, the aromatic compound benzaldehyde (metabolite 41) was identified here as a potential marker for caterpillar-infested *B. bassiana* endophytically colonized wild cotton plants (Figure 1; Supplementary Table 1) and is known to cause a strong response in herbivore natural enemies (Han and Chen, 2002). Similarly, we found 4-methyl-octane (metabolite 21), (E)-hex-2-en-1-ol (metabolite 22), and α-caryophyllene (metabolite 97) to be released by *B. bassiana* colonized wild cotton plants (Figure 1; Supplementary Table 1), which have been previously identified as potential attractants to natural enemies (Dickens, 1989, 1999; Han and Chen, 2002; Reddy and Guerrero, 2004). We also found that decanal (metabolite 71) and caryophyllene (metabolite 95) were released in larger quantities by endophytically colonized cotton plants than by control plants, whereas this effect of the fungal colonization was neutralized by caterpillar infestation (Supplementary Table 1). These two compounds are often emitted after herbivore damage and they have been reported as plant synomones that attract natural enemies (Gouinguen et al., 2001; Degen et al., 2004; Rasmann et al., 2005; Zhang et al., 2020). Furthermore, decanal (metabolite 71) and caryophyllene (metabolite 95), together with α-caryophyllene (metabolite 97) have also been reported as key plant compounds contributing to the attraction of natural enemies by cotton plants damaged by insect pests (Morawo and Fadamiro, 2016), whereas the role of these compounds in our multitrophic system remains unknown and will need a deeper study.

The emission of 2,6,6-trimethylbicyclo[3.1.1]hept-2-ene (= α-pinene) (metabolite 33) and 1H-indole (metabolite 78) have also previously been shown to increase upon caterpillar infestation of cotton plants (Sobhy et al., 2014); here we show that indole emission is higher in fungally challenged caterpillar infested plants (Supplementary Table 1). Indole does not have the above-mentioned synomone-like effect, and is considered a general insect repellent (Han and Chen, 2002; Schröder et al., 2015).

Regarding melon, we provide first evidence that 2-ethylhexyl nonyl sulfite (metabolite 14) can serve as a biomarker for endophytically colonized melon plants that are simultaneously infested by the cotton aphid. This compound has never previously been reported to be emitted by plants or fungi. Similar organic esters of sulfurous acids, more particularly mixed sulfite diesters of aliphatic or aromatic monohydroxy compounds and glycol ethers, are known as useful insecticides, particularly for mite control (Covey et al., 1969). A next step of the present research will explore whether or not the emission of this kind of compounds by *B. bassiana* endophytically colonized and pest-infested plants could have an insecticidal effect on pests together with the possible role of 6-Methyl-octadecane (metabolite 50), which was more emitted by endophytically colonized melon plants, as a herbivore-induced plant volatile, that my serve as herbivore repellent and natural enemy attractant (Turlings and Ton, 2006; Nisha and Kennedy, 2017).

Some other compounds that were found to be enhanced by *B. bassiana* endophytic colonization have previously been reported to play different roles mediating tritrophic interactions insect-plants-natural enemies. For instance, benzaldehyde is an ancestral and common compound emitted by plants and insects that has been reported also to act as a sex pheromone, aggregation pheromone, alarm pheromone, and is used as defense secretion in several insect taxa (Schiestl, 2010). Another compound found in caterpillar-infested and endophytically colonized wild cotton plants that has been reported as insect sex pheromone is (2Z,13E)-octadeca-2,13-dien-1-ol (metabolite 108) (Tonini et al., 1986; Islam et al., 2007; Yang et al., 2009; Ubaid et al., 2016). We found 1-iodo-2-methylundecane (metabolite 34), as dominant compounds in VOC blends of colonized melon plants that were not also infested by aphids, it has previously been identified as a floral volatile with potential impact on insect visitation (Wang et al., 2014).

We also identified several volatile compounds with known antifungal and antimicrobial activity. For instance, benzahaldehyde (metabolite 41) and (2Z,13E)-octadeca-2,13-dien-1-ol (metabolite 108), which were found mainly for wild cotton plants that were caterpillar-infested, as well as colonized by the fungus, are known to have antimicrobial activity in other systems (Schulz et al., 1995; Wilson et al., 1999; Teles et al., 2006; Wang et al., 2006; Li et al., 2008; Shao et al., 2009; Lin et al., 2012; Sun et al., 2014; Ullah et al., 2015; Zhou et al., 2016; de Amorim et al., 2019). For example, benzahaldehyde has a negative impact on conidial germination, fungal growth speed, and conidial production of the entomopathogenic fungus *Lecanicillium lecanii* (Zimm.) Zare & W. Gams (Lin et al., 2017). Moreover, the compounds 1-iodo-2-methylundecane (metabolite 34) and oxolan-2-one (metabolite 51), which we found in higher quantities in colonized melon plants, have been proposed to play a functional role in the chemical defense against microbial invasion (Cazar et al., 2005; Manilal and Idhayadhulla, 2014; Leena et al., 2016; Li et al., 2019). Hexadecan-4-yl 2,2,2-trifluoroacetate (metabolite 36) found in endophytically colonized melon plants with aphids, has been linked to antimicrobial activity against several bacterial and fungal species (Sarada et al., 2011; Managamuri et al., 2017; Mannaa and Kim, 2018). Finally, 6-methyl-octadecane (metabolite 50), identified in colonized melon plants, my provide resistance against phytopathogenic nematodes (Seenivasan, 2018). Both 1-iodo-2-methylundecane (metabolite 34) and 6-methyl-octadecane (metabolite 50) are bioactive compounds emitted by the entomopathogenic fungus *Paecilomyces lilacinus* (Thom) Samson (Ansari, 2019).

The enhanced production of the above compounds could be a possible explanation of the observed resistance against several plant phytopathogens when the plants are colonized by entomopathogenic fungi (Jaber and Ownley, 2018). It implies that endophytic entomopathogenic fungi produce or cause the production of secondary metabolites with inhibitory activity against mycelial growth, conidial germination, and germ-tube elongation of phytopathogenic fungi, as well as against plant virus replication and accumulation (Ownley et al., 2008, 2010; Jaber and Ownley, 2018).

In our experimental system, compounds previously reported to posse allelopathic effects on several weed species have been detected, whereas their specific origin and function in our multitrophic systems remains unraveled. This is the case for 2,7,10-trimethyldodecane (metabolite 63) that has been found to be related colonized wild cotton plants and melon plants infested with aphids (Li et al., 2016). In contrast, 6-methyl-octadecane (metabolite 50), found in colonized melon plants, has been related to the induction of seed germination (Vanitharani and Pandian, 2012; Vanitharani, 2014).

Some of the compounds found in the caterpillar-infested and endophytically colonized cotton plants had not previously been identified from plants, insects or microorganisms. This is the case for 1,1,2-trimethyl-3-(2-methylpropyl)-cyclopropane (metabolite 23) and which displayed higher overall concentrations in caterpillar-infested control plants compared to the caterpillar-infested colonized plants. Their effect on plant-insect interactions and plant-pathogen interactions should be investigated in future research. Indeed, it would also be interesting to see what effects the inducible volatile emissions may have on neighboring plants. Enhanced plant resistance mediated by VOCs will have further implications for crop protection (Brilli et al., 2019).

In summary, the present study confirms that the endophytic colonization of melon and wild cotton plants by the entomopathogenic fungus *B. bassiana* causes significant changes in the blend of volatile compounds emitted by the plants, also when they are infested by chewing or sap-sucking insects. These findings indicate an even greater potential of the use of entomopathogenic fungi as an ecologically safe strategy of biological pest control, as the enhanced volatile emissions may for example boost natural enemy attraction in agroecosystems, though further research is needed to address such a hypothesis.

## Data Availability Statement

The original contributions presented in the study are included in the article/Supplementary Material, further inquiries can be directed to the corresponding authors.

## Author Contributions

EQ-M, TT, and NG-M conceived and designed the study. LG, FG-S, NG-M, and AS-O did the experiments. JM, JM-R, and AS-O analyzed the data. EQ-M, NG-M, AS-O, JM, JM-R, and TT wrote the manuscript. All authors read and approved the manuscript before submission.

## Conflict of Interest

The authors declare that the research was conducted in the absence of any commercial or financial relationships that could be construed as a potential conflict of interest.

## Publisher's Note

All claims expressed in this article are solely those of the authors and do not necessarily represent those of their affiliated organizations, or those of the publisher, the editors and the reviewers. Any product that may be evaluated in this article, or claim that may be made by its manufacturer, is not guaranteed or endorsed by the publisher.
